# Abcès du coude révélant la maladie des griffes du chat: à propos d’un cas

**DOI:** 10.11604/pamj.2017.27.67.12427

**Published:** 2017-05-30

**Authors:** Mustafa Nkaoui, Ahmed El Bardouni, Omar Lazrek, Nasser Ibo, Fouad Zouaidia, Mohamed Kharmaz, Mohamed Elouadghiri, Omar Lamrani, Mustapha Mahfoud, Mohamed Saleh Berrada

**Affiliations:** 1 Service de Chirurgie Orthopédique et de Traumatologie, CHU Ibn Sina, Université Mohammad V Souissi, Rabat, Maroc; 2 Service d’Anatomie Pathologique, CHU Ibn Sina, université Mohammad VSouissi, Rabat, Maroc

**Keywords:** Maladie des griffes du chat, bartonella, rapport de cas, Cat-scratch disease, bartonella, case report

## Abstract

La maladie des griffes du chat (MGC) apparaît comme une cause fréquente de lymphadénopathie chronique bénigne chez l'enfant et l'adulte jeune. L'agent responsable de la maladie est Bartonella henselae. Les symptômes habituels sont une lymphadénopathie régionale associée à une fièvre. Nous rapportons une observation cliniquement atypique et potentiellement trompeuse de MGC, révélée par un abcès du coude chez une fille de 18 ans.

## Abstract (en)

Cat-scratch disease (CSD) is a common cause of chronic benign lymphadenopathy in the child and the young adult. Bartonella henselae is the agent responsible for this disease. Common symptoms include regional lymphadenopathy associated with fever. We report a clinically atypical and potentially misleading case of a 18-year old girl with CSD revealed by elbow abscess.

## Introduction

Décrite en 1950 par Robert Debré, la maladie des griffures du chat, ou lymphoréticulose bénigne d'inoculation est une infection bactérienne due à un petit bacille à Gram négatif, Bartonella henselae. Le chat en est le vecteur principal, qu'il peut transmettre à l'homme par griffade, morsure ou léchage. Elle atteint essentiellement l'enfant et l'adulte jeune (80%) des patients ont moins de 18 ans [1] et se manifeste habituellement par une ou plusieurs adénopathies chroniques dans le territoire de drainage de la zone d'inoculation. Dans près de la moitié des cas, des signes généraux sont associés [2]. Des aspects atypiques (environ 10% des cas) ont été récemment individualisés témoignant d'une diffusion systémique ou d'une localisation particulière. Nous rapportons une observation d'évolution favorable dont la présentation clinicoradiologique évoque un processus tumoral abcédé situé au niveau du bras. Cette localisation exceptionnelle s'ajoute aux formes pseudo tumorales rapportées jusqu'alors [3].

## Patient et observation

**Figure 1:**
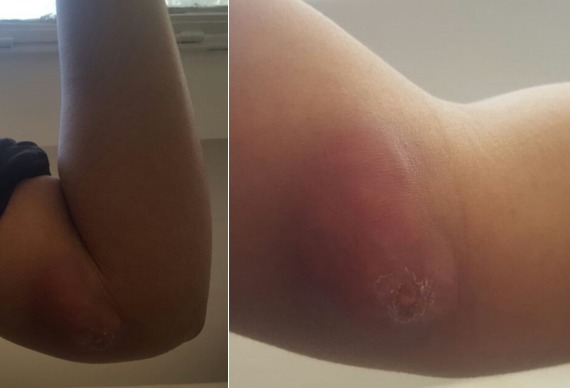
Image clinique montrant une masse inflammatoire de la face interne du coude gauche chez une patiente de 18 ans

**Figure 2:**
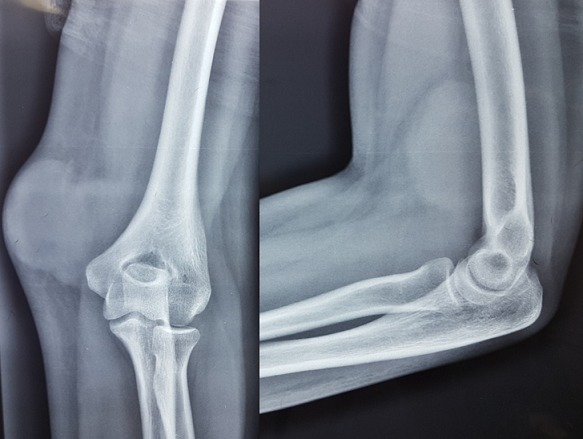
Radiographie du coude gauche face et profil: absence de lésion osseuse sous-jacente ou corps étranger

**Figure 3:**
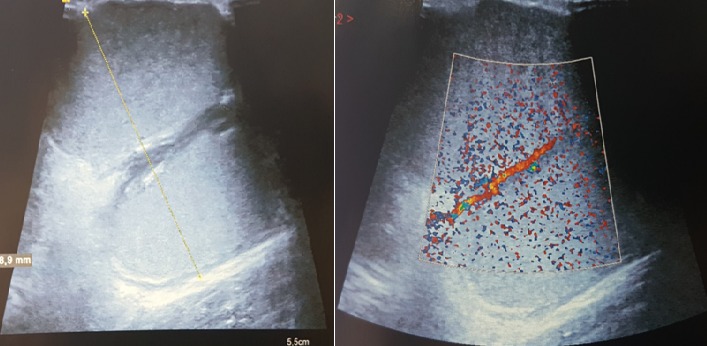
Échographie du coude: aspect bilobé, hétérogène de la masse, bien vascularisée au doppler

**Figure 4:**
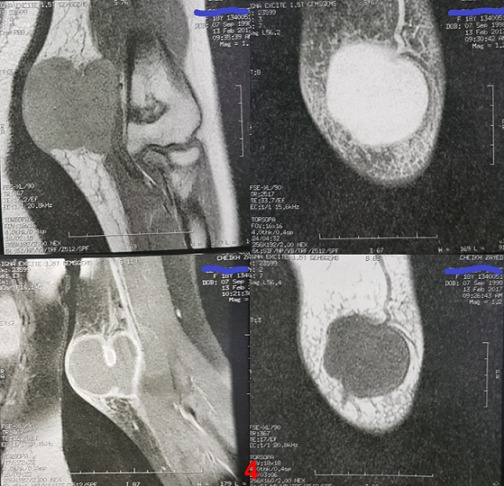
IRM du bras: collection hétérogène à contenu liquidien avec prise de contraste périphérique après injection évoquant un abcès

**Figure 5:**
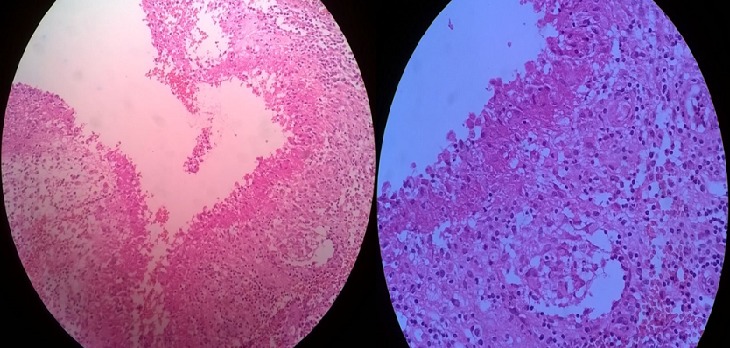
Aspect histologique compatible avec la maladie des griffes du chat: foyers inflammatoires granulomateux épithéliogigantocellulaires microabcédés

Patiente de 18 ans, sans antécédents pathologiques particuliers, qui s'est présenté aux urgences pour tuméfaction douloureuse de la face interne du coude gauche, sans notion de traumatisme ou piqure récente évoluant depuis environ un mois avec augmentation progressive de taille, dans un contexte de fébricule et conservation de l'état général, ayant justifié une prise d'anti-inflammatoires sans amélioration. L'examen clinique retrouvait une température à 37°, une tuméfaction de 7 cm de diamètre au niveau de la face interne de l'extrémité inférieure du bras gauche en regard du coude avec signes inflammatoires à type de rougeur ,chaleur locale associée à une petite fistule avec issu de sérosités purulentes à la pression, la masse était tendue douloureuse à la palpation de consistance molle fixe par rapport au plan superficiel mobile par rapport au plan profond (Figure 1). La mobilité du coude était conservée sans douleur et sans signes d'arthrite. L'examen vasculonerveux du membre supérieur gauche était sans particularité. Le reste de l'examen somatique a retrouvé des adénopathies satellites dans le territoire axillaire gauche. Le bilan biologique a montré un syndrome inflammatoire limite avec une leucocytose à 10 500/mm^3^ et une CRP à 24 mg/L. La radiographie standard du coude gauche était normale sans lésion osseuse et sans visualisation de corps étranger (Figure 2). L'échographie doppler du bras a montré une masse sous cutanée, hétérogène, bilobée, bien vascularisée (Figure 3). L'imagerie par résonance magnétique a objectivé une collection de 6 cm hétérogène à contenu liquidien avec prise de contraste périphérique après injection évoquant une formation abcédée (Figure 4). Un drainage chirurgical a été réalisé mettant en évidence un contenu purulent. L'analyse bactériologique était négative. L'étude histologique a montré un remaniement inflammatoire et granulomateux subaigu avec des cultures mycologiques et mycobactériologies négatives. Il n'existait pas de signes histologiques de malignité (Figure 5). Le diagnostic de la maladie des griffes du chat a été alors évoquée. La reprise de l'anamnèse a révélé effectivement une notion de contact fréquent avec un chat. Une première sérologie de Bartonella henselae, était limite et non significative. Une séroconversion a été notée avec une deuxième détermination, 20 jours après la première. Un traitement oral de 5 jours par l'azithromycine a été prescrit. Les symptômes et les adénopathies ont disparu progressivement après 2 mois.

## Discussion

Décrite pour la première fois par Debré en 1950 [4]. La maladie des griffes du chat est une affection fréquente et bénigne due à une bactérie intracellulaire facultative du groupe gamma des protéobactéries: Bartonella henselae. Son réservoir est le chat et son hôte est l'homme. Elle est transmise par la morsure ou les griffures d'un chat ou par ses puces. Le tableau clinique de la forme typique est l'apparition d'une adénopathie subaiguë, précédée d'une lésion d'inoculation et associée ou non à quelques signes généraux. Après une période d'incubation de trois à dix jours, 60 à 93% des patients développent une lésion d'inoculation au niveau du site de griffure initiale. Il s'agit d'une papule ou d'une pustule, plus ou moins prurigineuse, mesurant 3 à 5 mm qui peut persister de quelques jours à quelques mois [5]. Deux à trois semaines après, apparaît une adénopathie dans le territoire de drainage lymphatique du site d'inoculation. La localisation est par ordre de fréquence axillaire (51%), cervicale (28%), inguinale (16%) ou au coude (2 à 13%). Dans moins de 15% des cas, il s'agit d'adénopathies multiples dans le même territoire. Des formes pluriganglionnaires sont possibles mais plus rares (10%) ; elles sont considérées comme la conséquence d'inoculations multiples. Le syndrome ganglionnaire peut s'associer à des signes généraux: une fièvre dans un tiers des cas, une asthénie (30%), une anorexie (15%), des céphalées (14%), une dysphagie (7,6%), et une conjonctivite (3,7%) [6]. Les formes atypiques de la maladie des griffes du chat surviennent chez l'enfant immunodéprimé mais aussi immunocompétent. La maladie peut alors revêtir différents aspects : atteinte ophtalmologique (syndrome oculoganglionnaire de Parinaud) [7], systémique (Dans ce contexte, une atteinte neurologique, hépatosplénique ou osseuse peut exister) [8], ou parfois une forme pseudotumorale [3] ou abcédée comme celle rapportée dans notre observation. Il s'agit le plus souvent d'une masse localisée au niveau des parties molles épargnant généralement l'os. Ce qui pose un problème de diagnostic différentiel avec d'autres maladies plus graves telles que les mycobactérioses ou les maladies hématologiques. En effet, L'interrogatoire et l'examen physique de notre patiente n'a pas rapporté une lésion initiale de griffure ou d'inoculation qui peut nous orienter vers cette maladie. La confirmation paraclinique est plus difficile, à l'instar de notre observation.Les examens biologiques de routine ne sont pas spécifiques et ne présentent souvent aucune anomalie [9]. L'hyperleucocytose, est signalée de manière inconstante dans d'autres publications, avec une fréquence de 30 à 40%, et traduit généralement une forme plus sévère avec des signes généraux plus marqués ou une dissémination systémique [10]. Un syndrome inflammatoire souvent modéré est notifié.

La ponction ganglionnaire, lorsqu'elle est réalisable, permet d'obtenir un pus épais et souvent verdâtre. L'analyse histologique du pus ou de la biopsie ganglionnaire est évocatrice du diagnostic mais non spécifique. L'aspect histologique est celui d'un granulome pyoépithélioïde gigantocellulaire avec parfois une nécrose centrale. Conduisant l'anatomopathologiste à répondre « aspect compatible » avec le diagnostic de maladie des griffes du chat [11]. La sérologie est la méthode diagnostique la plus simple mais elle présente divers défauts responsables d'un manque de sensibilité et spécificité [12]. La date de réalisation du test est primordiale ; un test réalisé trop tôt peut être faussement négatif. La recherche d'une séroconversion ou d'une augmentation significative des titres des anticorps doit s'effectuer sur deux sérums à 15 jours d'intervalle [13]. Dans notre cas, nous avons pensé à refaire cette sérologie chez notre patiente vue que la première était non significative. La mise en évidence d'ADN de Bartonella henselae par PCR est la technique la plus sensible et surtout la plus spécifique. Elle peut être effectuée à partir de prélèvements bactériologiques issus de ponction ganglionnaire, d'adénectomie chirurgicale et/ou d'une lésion d'inoculation. Selon une étude rétrospective menée à Angers, l'association d'une sérologie et d'une PCR permettrait d'obtenir une sensibilité de 92% [11]. Il semble donc important d'utiliser les deux méthodes si l'on souhaite obtenir une certitude diagnostique. Le diagnostic est généralement retenu sur l'existence d'au moins trois critères parmi les quatre suivants: un contact avec un chat; une présentation clinique évocatrice et une évolution satisfaisante; l'absence d'autre cause retrouvée; la présence soit d'une sérologie positive, soit d'une amplification génique positive, soit d'un examen anatomopathologique évocateur [11]. Chez notre patiente, trois de ces critères nous ont permis de poser le diagnostic. L'évolution de la maladie des griffes du chat, qu'il s'agisse d'une forme typique ou atypique est généralement favorable et ne nécessite, dans la majorité des cas, qu'un traitement symptomatique. Les adénopathies persistent en moyenne de deux à quatre mois, plus rarement six à 24 mois. L'introduction d'une antibiothérapie est discutée car elle ne semble pas diminuer la durée d'évolution de la MGC. Néanmoins, elle peut diminuer plus rapidement le volume des adénopathies et réduire les signes généraux [14]. Bartonella henselae est sensible à plusieurs antibiotiques: l'azithromycine, la rifampicine, la ciprofloxacine, le cotrimoxazole et la gentamicne. Nous avons utilisé l'azithromycine, qui semble être le meilleur choix thérapeutique et qui a l'avantage d'une courte durée (500 mg à j1 puis 250 mg/jour de j2 à j5) [2,15]. Dans les formes sévères (adénopathies volumineuses, multiples et douloureuses) ou avec atteintes systémiques, la durée de traitement recommandée est d'un mois minimum; une bi-antibiothérapie est à discuter [14]. Dans les formes ganglionnaires abcédées ou fistulisées, le drainage chirurgical est indiqué. Par ailleurs, il n'existe pas de traitement préventif ni de vaccination disponible pour les chats. En cas de griffure, la conduite à tenir est un lavage abondant et une antisepsie de la plaie.

## Conclusion

La maladie des griffes du chat est une maladie bénigne en dehors de quelques localisations inhabituelles. Le diagnostic doit cependant être posé afin d'écarter d'autres maladies plus graves. Dans notre observation l'aspect clinique et radiologique de la masse sous-cutanée aurait pu correspondre à une lésion maligne. L'imagerie par résonance magnétique a évoqué un abcès. Devant une masse des parties molles touchant les membres ou la face et s'accompagnant d'adénopathies régionales, il paraît en effet légitime de rechercher un contact avec un chat afin d'orienter les investigations vers une maladie des griffes du chat.

## Conflits d’intérêts

Les auteurs ne déclarent aucun conflit d'intérêts.
